# Supplementary material for: Probiotic powder ameliorates colorectal cancer by regulating Bifidobacterium animalis, Clostridium cocleatum, and immune cell composition
Source: PLoS One. 2023 Mar 13;18(3):e0277155. doi: 10.1371/journal.pone.0277155 (PMC10010516; doi:10.1371/journal.pone.0277155)

Images of the original western blots

1.The following image represents represents western blot analysis shown in Fig 5

The First result (BAX)


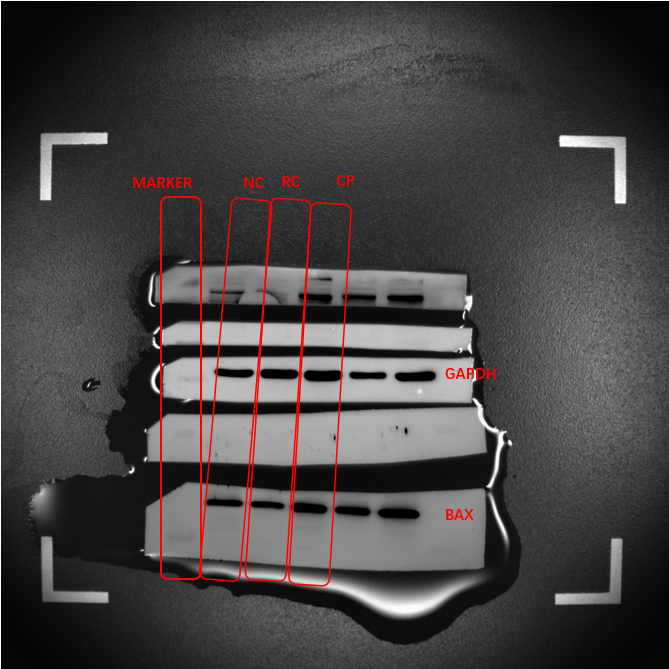


The second result (BAX)


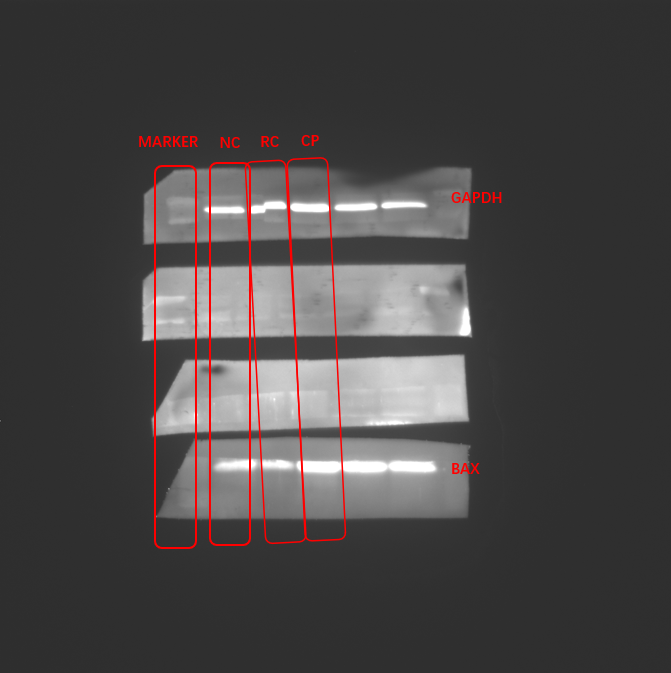


The third result (BAX)


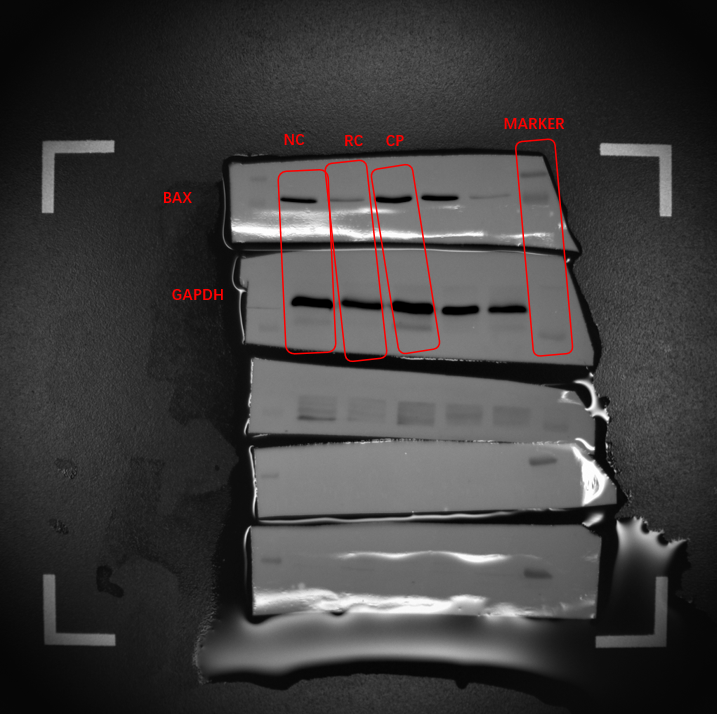


The fourth result (BAX)


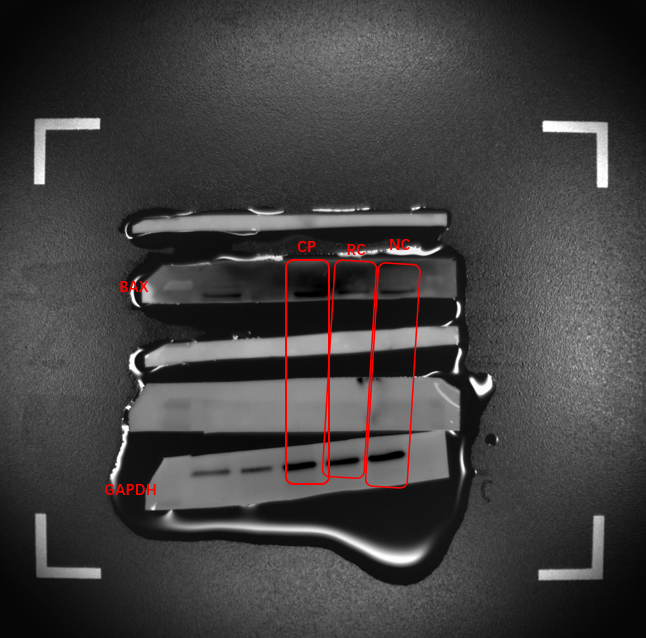


The First result (Bcl-2)


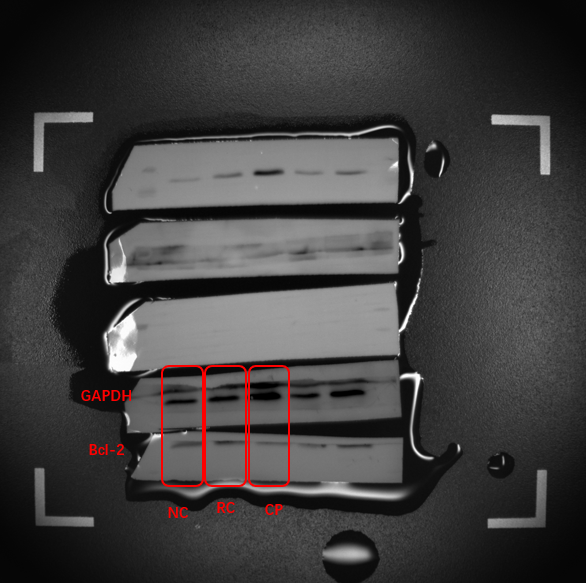


The second result(Bcl-2)


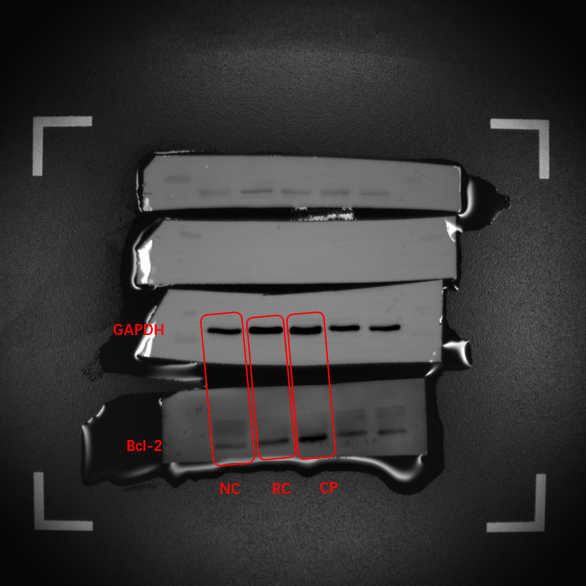


The third result(Bcl-2)


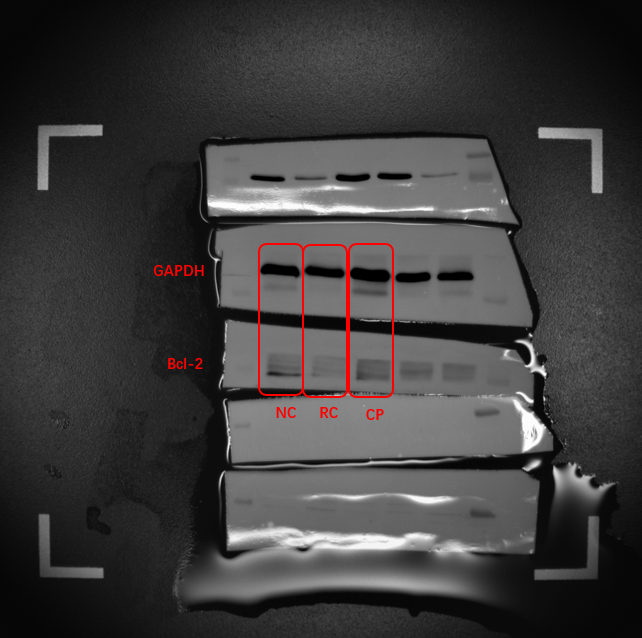

Supplement: S1 File — (DOCX) [file pone.0277155.s002.docx]
