# Supplementary material for: Probiotic powder ameliorates colorectal cancer by regulating Bifidobacterium animalis, Clostridium cocleatum, and immune cell composition
Source: PLoS One. 2023 Mar 13;18(3):e0277155. doi: 10.1371/journal.pone.0277155 (PMC10010516; doi:10.1371/journal.pone.0277155)
Supplement: Attachment — Submitted filename: Response to Reviewers.docx [file pone.0277155.s003.docx]

Dear editor and reviewer：

I am very happy to receive your decision. First of all, thank you for your interest and affirmation to my research. It is my honor to submit my manuscript to your Journal and I am very cherishing the opportunity to be accepted by your Journal. Therefore, I have made serious amendments to the questions you raised, and the specific answers will be displayed in the following reply form. I am very appreciated for your consideration and looking forward to your reply, hope everything goes well with your work and everything goes your way. As follows are the specific responses.

**Editor**

Thank you for giving us the opportunity to submit a revised draft for publication in the journal of PLOS ONE. The following are the changes we made based on your suggestion:

1. manuscript style requirements

We have modified the style of the article in accordance with the requirements of PLOS ONE, including figure style, table style, references and so on.

2. Information regarding the experiments involving animals

In the content of the “Mice” in the Methods part, we supplement the animal experiment and make sure that it includes the description of methods of sacrifice, efforts to alleviate suffering and so on.

3. Data Availability statement

In the data availability statement, we have specified where the minimal data set underlying the results described in our manuscript can be found, and we promise that all the minimal data in our articles will be fully available. The link in the Data Availability statement (https://www.ncbi.nlm.nih.gov/bioproject/PRJNA669487) is the Illumina sequencing raw data we uploaded to NCBI. In addition, we have uploaded a supporting Information files for study’s minimal underlying data. In addition, we have submitted all other results in the manuscript in the form of a supporting file, which is called “Minimal data set”

4. Uncropped and unadjusted images underlying all blot

We uploaded the original image without uncropped and unadjusted images. The file name is "Images of the original western blots"

5. Ethics statement

We have deleted the ethics statement in other parts of the article and only described the ethics statement in the methods section

**Reviewer #1**

Thank you for your affirmation to my work and your valuable suggestions, which has greatly improved the quality and accuracy of our manuscript. The following are the changes we made based on your suggestion:

1. Explain the relationships among cancer, microbiota, lymphocytes, and

BAX expression

We think this is an excellent suggestion, and we made changes to this suggestion, mainly in the description of the results, as well as the conclusion. We think Probiotic powder can ameliorate CRC by regulating gut microbiota (increased the abundance of *Bifidobacterium animalis* and reduced the abundance of C*lostridium cocleatum*). The changes of gut microbiota in turn regulate the immune environment of CRC mice, including attenuating CD4+ Foxp3+ Treg cells, promoting CD4+ IL-4+Th2 cells and inhibiting the expression of TIGIT in CD4+ IL-4+Th2 cells, promoting CD19+ GL-7+B cells. The above changes of immune cells induced effective anti-tumor immune response in CRC mice, which enhanced the ability of immune cells to kill tumor cells. As a result, the biological function of tumor cells has been changed, resulting in the high expression of pro-apoptotic proteins and then apoptosis. The number of apoptotic tumor cells increased, resulting in the inhibition of CRC.

2. The title of the manuscript

Thanks for your suggestions, we have refined the title, and now the title is “Probiotic powder ameliorates colorectal cancer by regulating Bifidobacterium animalis, Clostridium cocleatum, and immune cell composition ”

3. About references are cited in Methods and Results sections, figure

legends and the resolution of figures

Thanks for your suggestions. First of all, we make some changes to the description of the methods and results, and add references, such as Ref. 10-19. Secondly, we modified the figure legends and make a full description of the figure legends by referring to other manuscript. Finally, we adjusted the figures and improved the resolution of the figures.

4. In Results section, the authors should first explain the purpose of each

experiment, citing appropriate references.

Thank you for pointing this out, we have added the purpose of each experiment in the results section and inserted relevant references, such as references 13-19.

5. The issue in Fig. 1A-C

Your suggestion really means a lot to us. We have added different colors of arrows to indicate different tissue damages, such as the red arrow represents the normal goblet cells, the green arrow represents the crypt abscess, the blue arrow represents inflammatory necrosis, and the black arrow represents inflammatory cell infiltration.

6. The issue in Fig. 4

Thank you for your positive comments. In order to explore the effect of probiotic powder on immunity of mice by regulating gut microbiota, we are not limited to the analysis of these specific cells. In addition to these cells, we also analyzed CD11C^+^MHCII^+^DC cells, CD4^+^,CXCR3^+^Th1 cells and CD4^+^,CCR6^+^Th17 cells. But in these cells, only CD4^+^ Foxp3^+^ Treg cells, CD4^+^ IL-4^+^ Th2 cells and CD19^+^ GL-7^+^ B cells changed significantly. Therefore, we believe that Probiotic powder play a role mainly by affecting these three immune cells in CRC mouse model.

7. The issue in Fig. 5

Thank you for pointing this out, we have made a supplementary description in the results and figure legends, the extracted protein is a protein from mouse colorectal cancer tissue, and then the expression of BAX in this tissue was detected. In addition, we fell sorry for our carelessness, in our resubmitted manuscript, the molecular weight of protein has been indicated as kDa.

**Reviewer #2**

We fell great thanks for your professional review work on our article. As you are concerned, there are several problems that need to be addressed. According to your nice suggestions, we have made extensive corrections to our provious draft, the detalied corrections are listted below.

1. The issue in Fig 1

Thank you for pointing this out, after careful consideration, we adjusted Fig 1E. from the comparison of the maximum tumor volume between RC and CP to the average tumor volume comparison between RC and CP，through the analysis, there was significant difference between the two groups (P < 0.05). The average tumor volume of different mice and the number of mice in each group were shown in Table2.

2. Whether the probiotic powder reduced or enhanced the degree of

inflammation induced by DSS.

We sincerely appreciate you valuable communts. In determining the effect of probiotic powder on inflammation caused by DSS, we mainly judged by HE staining, HE shows that probiotic powder can significantly reduced the degree of inflammation of CRC. We are sorry that we did not consider the weight change to reflect the degree of inflammation at the same time. Thank you very much for your suggestion. We will include this factor in later studies.

3. About probiotic powder

Thank you for pointing this out, the probiotic powder used in this study is our own optimized mixture, mainly based on the previous experimental results, the use standards of related additives, and the guidance of professional instructors (Professor Fan Yanli of Food discipline in Ningxia University, Professor Yang Jianhong, doctoral supervisor of Pharmacy in Ningxia Medical University, and Xia Yuhan, director of the Department of Nutrition in General Hospital of Ningxia Medical University).

4. Provide the molecular weight of DSS used in this study.

Thanks for your suggestions, we have added the molecular weight of DSS and the molecular weight of AOM in the Methods. As you can see, Azoxymethane(CAS No.:[25843-45-2](https://www.sigmaaldrich.cn/CN/zh/search/25843-45-2?focus=products&page=1&perpage=30&sort=relevance&term=25843-45-2&type=cas_number), molecular weight :74.08) and dextran sulfate sodium salt(CAS No.:9011-18-1, molecular weight : ~40,000) .

5. Grammatical editing problem.

As soon as we received the decision, we submitted the manuscript to Charlesworth Author Services （CAS）for correct English language usage, grammar, punctuation and spellings. We have submitted a version of the manuscript edited by CAS (file name is “Revised Manuscript by CWS”) and an Editorial certificate (file name “CWS_Editorial_Certificate”)
