# Supplementary material for: Probiotic powder ameliorates colorectal cancer by regulating Bifidobacterium animalis, Clostridium cocleatum, and immune cell composition
Source: PLoS One. 2023 Mar 13;18(3):e0277155. doi: 10.1371/journal.pone.0277155 (PMC10010516; doi:10.1371/journal.pone.0277155)
Supplement: Attachment — Submitted filename: Response to Reviewers.docx [file pone.0277155.s004.docx]

Dear editor and reviewer：

Thank you for giving us the opportunity to resubmit a revised draft of the manuscript “Probiotic powder ameliorates colorectal cancer by regulating *Bifidobacterium animalis*, *Clostridium cocleatum*, and immune cell composition” for publication in the Journal of PLOS ONE. We appreciate the time and effort that the editor and reviewers dedicated to providing insightful comments and valuable suggestions to our paper. I am very appreciated for your consideration and looking forward to your reply, hope everything goes well with your work and everything goes your way. As follows are the specific responses.

**Editor**

Thank you for giving us the opportunity to submit a revised draft for publication in the journal of PLOS ONE. The following are the changes we made based on your suggestion:

1. Please review your reference list to ensure that it is complete and

correct.

Our reply:

Thank you for pointing this out, we checked our reference list, the 23rd reference was incomplete and we re-cited it to make sure the references were complete and correct. In addition, our references do not contain articles that have been retracted, and all references are searchable on Pubmed.

**Reviewer #1**

We feel great thanks for your professional review work on our articles. As you are concerned, there are several problems that need to be addressed. Based on your nice suggestions, we have made extensive corrections to our previous manuscript, the detalied corrections are listted below.

1. In Fig. 4, the authors need to analyze Th1 cells, Th17 cells, and CD8+

T cells.

Our reply:

Thank you for pointing this out, this suggestion really means a lot to us. In our experimental design, we wanted to investigate the mechanism of the role of key gut microbiota in colorectal cancer by affecting Treg cells. Since Treg cell mainly plays an immunosuppressive function in cancer, we examined the changes of various types of effector T cells (CD4^+^ T cells), including Th1 cells, Th2 cells and Th17 cells, while CD8^+^ T cells were not considered. Ultimately, Th2 cells were found to play a major function. We apologize for not including other effector T cells with no statistically significant changes in Figure 4. Thanks to your valuable suggestion, we have added Th1 cells and Th17cells to Figure 4 (Fig. 4C), and described accordingly in the results and methods. As shown below:

2. Many letters in Fig. 2A, Fig. 2B, Fig. 3B, Fig. 4A, Fig. 4B, Fig. 4C

are not visible because of their inappropriate size and poor resolution.

Our reply:

Thanks for your suggestions, we have resized and sharpened all the figures in the article including Fig. 2A, Fig. 2B, Fig. 3B, Fig. 4A, Fig. 4B and Fig. 4C. The resolution and sharpness of the figures have been improved.

Thanks again for your reconsideration of publishing our manuscript in your journal. We are looking forward to hearing from you soon!

With best wishes

Sincerely
