# Supplementary material for: Probiotic powder ameliorates colorectal cancer by regulating Bifidobacterium animalis, Clostridium cocleatum, and immune cell composition
Source: PLoS One. 2023 Mar 13;18(3):e0277155. doi: 10.1371/journal.pone.0277155 (PMC10010516; doi:10.1371/journal.pone.0277155)
Supplement: Attachment — Submitted filename: Response to Reviewers.docx [file pone.0277155.s005.docx]

Dear editor and reviewer：

Thank you for your letter and for the reviewers’ comments. We really appreciate the reviewers for your valuable comments and we have studied comments carefully and revised the manuscript. We hope the revised manuscript could be acceptable for publication in the Journal of PLOS ONE. The responds to the reviewer’s comments are as flowing, thank you in advance for your time and effort!

1.The first comments of Reviewer :The authors should analyze CD8^+^ T cells, because the study deals with cancer.

Our reply:

Thank you for pointing this out, we have analyzed CD8^+^ T cells, including the changes of IFN-γ^+^ CD8^+^ T cells and we added the results to Fig 4B, and described accordingly in the results , discussion, conclusion and methods. As shown below.

CD8+ T cells are the main force of the anti-tumour immune response, which clear tumors through a variety of mechanisms. IFN-γ secreted by CD8+T cells has anti-tumor activity and can control tumor growth. In addition, IFN- γ can enhance antigen presentation and inhibit tumor angiogenesis, which also plays an important role in cancer immune surveillance. Therefore, in this study, as the Reviewer said, it is very important to analyze CD8^+^ T cells.

As shown in Fig 4B, the number of IFN-γ^+^ CD8^+^ T cells was significantly reduced in the RC group compared with that in the NC group, intervention with the probiotic powder significantly reversed this decrease, suggesting that the probiotic powder promote the secretion of IFN-γ by CD8^+^ T cells to exert anti-tumour effects.

Based on the results of our analysis, our conclusions are as follows: In summary, we demonstrated that our probiotic powder ameliorated CRC by regulating *Bifidobacterium animalis*, *Clostridium cocleatum*, inhibiting Treg cells, promoting the number of IFN-γ^+^ CD8^+^ T cells, increasing Th2 cell abundance, inhibiting the expression of TIGIT in Th2 cells, and increasing B cell abundance in the immune microenvironment of CRC, resulting in increased expression of BAX in CRC. These results indicated that this probiotic powder has therapeutic potential for CRC.

2.The second comments of Reviewer :Most letters are still not visible in Figures.

Our reply:

In order to make the figures clearer, we have carefully revised the figures again. The resolution of the modified figures is 300dpi, and the dimensions, file size of the figures have been revised in strict accordance with the requirements of of PLOS ONE. In addition, we have uploaded our figure files to the PACE to ensure that our figures meet PLOS requirements.

We tried our best to revised the manuscript, we appreciate for Editors and Reviewers’ warm work earnestly, and hope that the correction will meet with approval. Once again thank you very much for you.

Best regards

Sincerely yours
