# Supplementary material for: Probiotic powder ameliorates colorectal cancer by regulating Bifidobacterium animalis, Clostridium cocleatum, and immune cell composition
Source: PLoS One. 2023 Mar 13;18(3):e0277155. doi: 10.1371/journal.pone.0277155 (PMC10010516; doi:10.1371/journal.pone.0277155)
Supplement: Attachment — Submitted filename: Response to Reviewers.docx [file pone.0277155.s006.docx]

Dear editor and reviewer：

Thank you for your letter and for the reviewers’ comments. We really appreciate the reviewers for your valuable comments and we have followed comments carefully and revised the manuscript. We hope the revised manuscript could be acceptable for publication in the Journal of PLOS ONE. The responds to editor and the reviewer’s comments are as flowing, thank you in advance for your time and efforts!

**Editor**: Please review your reference list to ensure that it is complete and correct.

Our reply:

Thank you for pointing this out, we have checked our references and ensured that its were completed and corrected. In addition, our references do not contain any articles that have been retracted, and all references are searchable on Pubmed.

**Reviewer #1:**Because I still do not see many letters in Figures, I would like to allow the editor or editorial office to make a decision.

Our reply:

We fell great thanks for the reviewer’s professional review work on our article. As the reviewer’s concerned, there still some letters are too small in Figures, we made adjustment again to our Figures. The letters in revised Figures are now of a higher quality and the resolution, dimensions, file size of the figures have been revised in strict accordance with the requirements of PLOS ONE. In addition, we have uploaded our figure files to the PACE to ensure that our Figures meet PLOS requirements. The following are the specific changes:

1.The letters in Fig 2A , Fig 2B, 3C are fully enlarged. Take Fig 2A as an example, the revised Figure is as follows:

2. AS Fig 3A and Fig 3B have too much contents, resulting in blurred letters. We make appropriate adjustments to Fig 3A and Fig 3B, and divided Fig 3A into two parts, named Fig 3A and Fig 3B respectively. The contents of Fig 3B is divided into four parts, which are named as Fig 3C, Fig 3D, Fig 3E and Fig 3F respectively. The adjusted Figures are clearer and the letters are visible. Take Fig 3A as an example:

3. The letters of Fig 4A , Fig 4B, Fig 4C , Fig 4D and Fig 4E are not clear due to the improper combination of the Figures. Therefore, we adjusted the way in which these Figures are combined. Take Fig 4E as an example, the adjusted image is as follows:

The Figures modification made the letters much clearer, the contents of the Figures has not changed, and does not affect the structure and description of our manuscript.

We tried our best to revised the manuscript, we appreciate to Editors and Reviewers’ hard work, and hope that the corrections could meet you requirement. Once again thank you very much for your nice patients.

Best regards

Sincerely yours
